# Diagnostic performance of standard breast MRI compared to dedicated axillary MRI for assessment of node-negative and node-positive breast cancer

**DOI:** 10.1007/s00330-020-06760-6

**Published:** 2020-03-27

**Authors:** Sanaz Samiei, Marjolein L. Smidt, Sigrid Vanwetswinkel, Sanne M. E. Engelen, Robert-Jan Schipper, Marc B. I. Lobbes, Thiemo J. A. van Nijnatten

**Affiliations:** 1 Department of Surgery, Maastricht University Medical Center+, P.O. Box 5800, 6202 AZ Maastricht, The Netherlands; 2 Department of Radiology and Nuclear Medicine, Maastricht University Medical Center+, Maastricht, The Netherlands; 3 GROW – School for Oncology and Developmental Biology, Maastricht University, Maastricht, The Netherlands; 4 Department of Surgery, Catharina Hospital, Eindhoven, The Netherlands; 5 Department of Medical Imaging, Zuyderland Medical Center, Sittard-Geleen, The Netherlands

**Keywords:** Breast cancer, Axilla, Lymph node, Metastasis, Magnetic resonance imaging

## Abstract

**Objectives:**

To investigate whether breast MRI has comparable diagnostic performance as dedicated axillary MRI regarding assessment of node-negative and node-positive breast cancer.

**Methods:**

Forty-seven patients were included. All had undergone both breast MRI and dedicated axillary MRI, followed by surgery. All included breast MRI exams had complete field of view (FOV) of the axillary region. First, unenhanced T2-weighted (T2W) and subsequent diffusion-weighted (DW) images of both MRI exams were independently analyzed by two breast radiologists using a confidence scale and compared to histopathology. ADC values were measured by two researchers independently. Diagnostic performance parameters were calculated on a patient-by-patient basis.

**Results:**

T2W breast MRI had the following diagnostic performance: sensitivity of 50.0% and 62.5%, specificity of 92.3%, PPV of 57.1% and 62.5%, NPV of 90.0% and 92.3%, and AUC of 0.72 for reader 1 and 0.78 for reader 2. T2W dedicated axillary MRI had the following diagnostic performance: sensitivity of 37.5% and 62.5%, specificity of 82.1% and 92.3%, PPV of 44.6% and 50.0%, NPV of 87.8% and 91.4%, and AUC of 0.65 for reader 1 and 0.73 for reader 2. In both evaluations, addition of DW images resulted in comparable diagnostic performance. For both breast MRI and dedicated axillary MRI, there was no significant difference between mean ADC values of benign and malignant lymph nodes.

**Conclusions:**

T2W breast MRI with complete FOV of the axillary region has comparable diagnostic performance as T2W dedicated axillary MRI regarding assessment of node-negative and node-positive breast cancer. Optimization of T2W breast MRI protocol by including a complete FOV of the axillary region can, therefore, be recommended in clinical practice.

**Key Points:**

*• Breast MRI with complete field of view of the axillary region has comparable diagnostic performance as dedicated axillary MRI regarding assessment of node-negative and node-positive breast cancer.*

*• Optimization of breast MRI protocol by including a complete field of view of the axillary region is recommended in clinical practice.*

*• For both breast MRI and dedicated axillary MRI, DW imaging (including ADC measurements) is of no added value.*

## Introduction

In breast cancer, the presence and extent of axillary lymph node metastases is an important prognostic indicator and helps in determining the optimal treatment plan [1–4]. Accurate assessment of axillary lymph node involvement, therefore, plays a pivotal role in breast cancer treatment. Over the past years, the surgical staging procedures of the axilla have evolved from routine axillary lymph node dissection (ALND) toward less extensive procedures, such as the sentinel lymph node biopsy (SLNB).

Parallel to surgical advances, the imaging techniques for axillary lymph node staging have improved and become increasingly useful as a non-invasive diagnostic imaging modality to assess the axillary lymph node status. This is important since accurate preoperative axillary imaging can contribute to a more patient-tailored treatment strategy regarding axillary surgery. In clinical practice, breast magnetic resonance imaging (MRI) is predominantly used in the preoperative setting to evaluate (initial) tumor extent [5–7]. Previous studies suggested promising results of breast MRI for assessing the axillary lymph node status [8, 9]. Breast MRI enables radiologists to simultaneously assess the breast tumor and axillary lymph nodes in the same field of view (FOV) if the axillary region is completely visualized with an acceptable signal-to-noise ratio (SNR). However, in 40% of the standard breast MRI exams, no complete FOV of the axillary region is planned, limiting the assessment of extensive axillary lymph node involvement in the upper part of the axilla [10]. Consequently, dedicated axillary MRI has been investigated to improve axillary lymph node imaging by representing the complete axillary region. Several studies have suggested that using a dedicated axillary surface coil increases the diagnostic performance of MRI for assessing the axillary lymph node status [11–15]. However, these results were based on studies, including patients that underwent only one of the two imaging protocols, not both.

To our knowledge, no previous study has compared standard breast MRI with dedicated axillary MRI within a single cohort of breast cancer patients who had undergone both MRI exams. Similar performance of axillary lymph node assessment on standard breast MRI, as opposed to dedicated axillary MRI, can make broad implementation possible given the existing breast MRI protocols. This study aimed to investigate whether standard breast MRI has a comparable diagnostic performance with that of dedicated axillary MRI regarding the assessment of node-negative and node-positive breast cancer using unenhanced T2-weighted (T2W) and diffusion-weighted (DW) images.

## Materials and methods

### Patient population

All patients with histopathologically confirmed invasive breast cancer between August 2012 and December 2014, who had undergone both standard breast MRI and dedicated axillary MRI followed by either SLNB or ALND, were considered for inclusion. Exclusion criteria were neoadjuvant systemic therapy (NST) before axillary surgery and breast MRI exams with incomplete FOV or poor SNR of the axillary region. The local medical ethics committee waived the necessity to acquire informed consent due to the retrospective study design.

### MRI acquisition

The breast MRI exams were performed with three different 1.5-T and 3.0-T scanners (Intera, Ingenia, and Achieva, Philips Healthcare), using a dedicated bilateral 16-channel breast coil with the patient in the prone position. The imaging protocol of 1.5-T breast MRI consisted of the following: (1) unenhanced three-dimensional (3D) T2W turbo spin-echo sequence without fat suppression (pixel size, 0.87 × 0.87 mm; repetition time (TR), 2000 ms; echo time (TE), 222 ms; echo train length, 92; flip angle, 90°; slice thickness, 2.0 mm); (2) contrast-enhanced T1W sequence; and (3) DW imaging sequence with fat suppression (*b*-values of 0, 150, and 800 s/mm^2^; pixel size, 1.28 × 1.28 mm; TR, 9670 ms; TE, 89 ms; echo train length, 68; flip angle, 90°; slice thickness, 3.0 mm). The imaging protocol of 3.0-T breast MRI consisted of the following: (1) unenhanced two-dimensional (2D) T2W turbo spin-echo sequence without fat suppression (pixel size, 0.59 × 0.59 mm; TR, 5294 ms; TE, 100 ms; echo train length, 27; flip angle, 90°; slice thickness, 2.0 mm); (2) contrast-enhanced T1W sequence; and (3) DW imaging sequence with fat suppression (*b*-values of 0, 150, and 800 s/mm^2^; pixel size, 1.25 × 1.25 mm; TR, 8683 ms; TE, 51 ms; echo train length, 43; flip angle, 90°; slice thickness, 3.0 mm). For standard breast MRI, the anatomic confines for an adequate FOV were between the humeral head and xiphoid process of the sternum. The dedicated axillary MRI exams were performed with a 3.0-T scanner (Achieva, Philips Healthcare), using a 32-channel cardiac coil with the patient in the supine position and ipsilateral arm elevated. The imaging protocol of dedicated axillary MRI consisted of the following: (1) unenhanced 3D T2W turbo spin-echo sequence without fat suppression (pixel size, 1.25 × 1.25 mm; TR, 2000 ms; TE, 155 ms; echo train length, 51; flip angle, 90°; slice thickness, 2.5 mm); (2) contrast-enhanced T1W; and (3) DW imaging sequence with fat suppression (*b*-values of 0, 500, and 800 s/mm^2^; pixel size, 1.38 × 1.38 mm; TR, 2110 ms; TE, 52 ms; echo train length, 71; flip angle, 90°; slice thickness, 3.0 mm). For dedicated axillary MRI, the anatomic confines for an adequate FOV were between the humeral head and inferior border of the scapula. The apparent diffusion coefficient (ADC) maps were automatically constructed for all DW images using the built-in MR software.

### Image analysis

The breast and dedicated axillary MRI exams were analyzed by two dedicated breast radiologists independently with significant experience in breast imaging (M.B.I.L. [reader 1] and S.V. [reader 2] with 11 and 8 years of experience, respectively). Comparable with clinical practice, the radiologists were aware of the laterality of the breast tumor and the clinical tumor size assessed by MRI. However, the histopathological outcome was not provided. For qualitative assessment, first, the ipsilateral axillary lymph nodes were assessed on the unenhanced T2W images and subsequently on the DW images of the standard breast MRI and dedicated axillary MRI exams separately. Both readers scored the axillary lymph nodes on the MRI exams using a 5-point confidence scale, ranging from 0 (no lymph nodes) to 4 (definitely malignant) [16]. The additional information from the DW images was used to adjust the score of the axillary lymph nodes based on the T2W images. If the DW image was unavailable or of poor image quality, the score remained unchanged. Characteristics of a malignant lymph node were based on size and morphologic features including irregular margins, inhomogeneous cortex, perifocal edema, asymmetry, loss of fatty hilum, and/or the absence of chemical shift artifact [16, 17]. For quantitative assessment, the DW images were analyzed by two researchers (S.S. [reader 3] and T.J.A.v.N. [reader 4]) dedicated to axillary lymph node imaging. Both readers were blinded to the histopathological outcome. High signal intensity area in the ipsilateral axilla on the DW images was detected and compared with the T2W images to evaluate whether or not it was an axillary lymph node. If multiple lymph nodes were detected, the lymph node with the longest axis was identified [18, 19]. A region of interest (ROI) was manually drawn on the DW images at *b* = 800 s/mm^2^ on one representative slice and then copied to the corresponding ADC map [20, 21]. The whole lymph node region with evidently high signal intensity was delineated in the case of subcentimeter lymph nodes, and in larger lymph nodes only the cortex was delineated avoiding the (fatty) hilum and surrounding tissue [22]. After the delineations, a consensus meeting was held to confirm the lymph node’s delineation between the two readers. Subsequently, both readers independently measured the mean ADC of the largest lymph node. The quantitative assessment was performed in OsiriX (version 10.0, Pixmeo SARL).

### Histopathological analysis

The lymph nodes obtained by axillary surgery were recorded as benign, isolated tumor cells (≤ 0.2 mm and/or < 200 cells in a single histological cross section), micrometastasis (0.2 ≤ 2.0 mm), or macrometastasis (> 2.0 mm) [23]. The isolated tumor cells and the micrometastases were considered negative, and macrometastases were considered positive axillary lymph nodes [24].

### Statistical analysis

Median and interquartile range (IQR) were given if patient characteristics were not normally distributed. On the confidence scale, the lymph nodes with the scores 0–2 were categorized as benign and lymph nodes with the scores 3–4 were categorized as malignant. Histopathology of the axillary surgery, SLNB or ALND, served as the gold standard. Diagnostic performance parameters of T2W images and T2W with DW images were calculated for both standard breast MRI and dedicated axillary MRI (sensitivity, specificity, positive predictive value (PPV), negative predictive value (NPV), and area under the receiver operating characteristic (ROC) curve (AUC)). The diagnostic performance parameters were presented with 95% confidence intervals (CIs). The nonparametric DeLong test was used to calculate the comparison between two AUCs [25]. Two-sided p values of <  0.05 were considered statistically significant. The mean ADC was compared between benign and malignant lymph nodes using the Wilcoxon signed-rank test. For the quantitative analysis, the intraclass correlation coefficient was calculated between readers 3 and 4. The difference between readers 3 and 4 in the ADC measurement and 95% limits of agreement were computed for standard breast MRI and dedicated axillary MRI. Statistical analyses were performed by using R project software (version 3.5.1, R Foundation for Statistical Computing) and Statistical Package for the Social Sciences software (version 25, IBM).

## Results

A total of 70 patients had undergone both standard breast MRI and dedicated axillary MRI. Twelve patients were excluded who had been treated with NST before axillary surgery. Eleven patients were excluded because of breast MRI exams with incomplete FOV of the axillary region. For final analyses, 47 patients (median age, 59 years; IQR, 51–66 years) were included. Thirty-nine (83.0%) patients had benign axillary lymph nodes at final pathology and 8 (17.0%) patients had malignant axillary lymph nodes. The median size of macrometastases was 11.5 mm (IQR, 8.0–25.5 mm). Patient characteristics are summarized in Table 1.

**Table 1:** Patient characteristics

Variable	Patients (*n* = 47)
Age (years) (median; IQR)	59 (51–66)
Clinical tumor size (mm) (median; IQR)	19 (13–28.5)
Clinical tumor stage (%)	
T1	25 (53.2)
T2	21 (44.7)
T3	1 (2.1)
Tumor type (%)	
Ductal	34 (72.3)
Lobular	7 (14.9)
Mixed ductal and lobular	3 (6.4)
Other*	3 (6.4)
Tumor grade (%)	
1	14 (29.8)
2	27 (48.9)
3	10 (21.3)
Receptor status (%)	
ER+HER2-	38 (80.9)
ER+HER2+	4 (8.5)
ER-HER2+	1 (2.1)
Triple negative	4 (8.5)
Breast surgery (%)	
Breast-conserving surgery	25 (53.2)
Mastectomy	22 (46.8)
Axillary surgery (%)	
SLNB	43 (91.5)
ALND	4 (8.5)

*Abbreviations*: *IQR*, interquartile range; *ER*, estrogen receptor; *PR*, progesterone receptor; *HER2*, human epidermal growth factor receptor 2; *SLNB*, sentinel lymph node biopsy; *ALND*, axillary lymph node dissection

*Other tumor types: adenoid cystic carcinoma, mucinous carcinoma, encapsulated papillary carcinoma

The T2W breast MRI had the following diagnostic performance: sensitivity of 50.0% and 62.5%, specificity of 92.3%, PPV of 57.1% and 62.5%, NPV of 90.0% and 92.3%, and AUC of 0.72 for reader 1 and 0.78 for reader 2. The addition of DW images resulted in comparable sensitivity (50.0%), specificity (92.3% and 94.9%), PPV (57.1% and 66.7%), NPV (90.0% and 90.2%), and AUC for reader 1 (0.73) and reader 2 (0.72). The comparison between the AUC values of T2W breast MRI and with the addition of DW images for reader 1 and reader 2 separately had a *p* value of 0.67 and 0.33, respectively. Two of the standard breast MRI exams had no DW images, and nine DW images had poor image quality assessed by the radiologists.

**Fig. 1:**
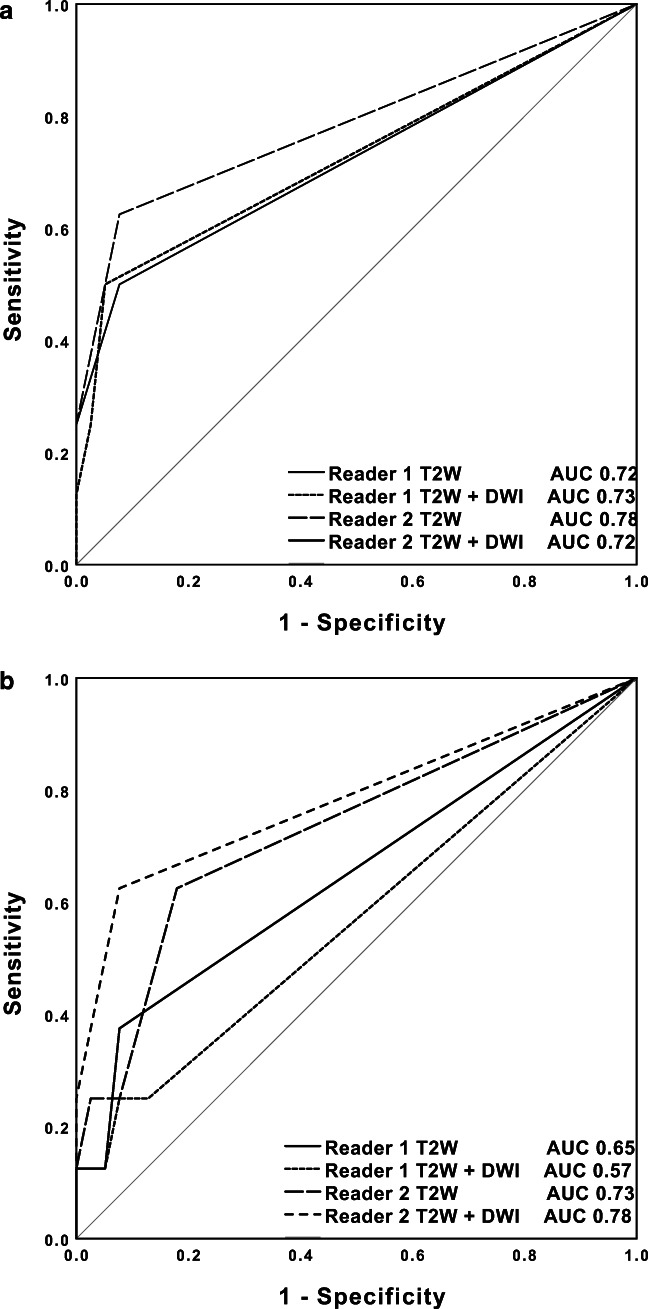
**a** Receiver operating characteristic curves show a qualitative assessment of T2-weighted (T2W) and T2W with diffusion-weighted (DW) standard breast MR images for readers 1 and 2. **b** Receiver operating characteristic curves show a qualitative assessment of T2W and T2W with DW dedicated axillary MR images for readers 1 and 2

The T2W dedicated axillary MRI had the following diagnostic performance: sensitivity of 37.5% and 62.5%, specificity of 82.1% and 92.3%, PPV of 44.6% and 50.0%, NPV of 87.8% and 91.4%, and AUC of 0.65 for reader 1 and 0.73 for reader 2. The addition of DW images resulted in comparable sensitivity (25.0% and 62.5%), specificity (87.2% and 92.3%), PPV (28.6% and 62.5%), NPV (85.0% and 92.3%), and AUC for reader 1 (0.57) and reader 2 (0.78). The comparison between the AUC values of T2W dedicated axillary MRI and with the addition of DW images for reader 1 and reader 2 separately had a *p* value of 0.21 and 0.068, respectively. One of the dedicated axillary MRI exams had no DW image. The results of the diagnostic performance parameters of T2W and T2W with DW images can be found in Table 2 and the ROC curves are presented in Fig. 1.

**Table 2:** Diagnostic performance of standard breast MRI with complete FOV and dedicated axillary MRI

	Reader 1		Reader 2	
	T2W	T2W with DW imaging*	T2W	T2W with DW imaging*
Standard breast MRI (*n* = 47)				
Sensitivity	50.0% (4/8)	50.0% (4/8)	62.5% (5/8)	50.0% (4/8)
	[15.7–84.3]	[15.7–84.3]	[24.5–91.5]	[15.7–84.3]
Specificity	92.3% (36/39)	94.9% (37/39)	92.3% (36/39)	92.3% (36/39)
	[79.1–98.4]	[82.7–99.4]	[79.1–98.4]	[79.1–98.4]
PPV	57.1% (4/7)	66.7% (4/6)	62.5% (5/8)	57.1% (4/7)
	[18.4–90.1]	[22.3–95.7]	[24.5–91.5]	[18.4–90.1]
NPV	90.0% (36/40)	90.2% (37/41)	92.3% (36/39)	90.0% (36/40)
	[76.3–97.2]	[76.9–97.3]	[79.1–98.4]	[76.3–97.2]
AUC	0.72	0.73	0.78	0.72
	[0.53–0.92]	[0.54–0.92]	[0.60–0.97]	[0.53–0.91]
Dedicated axillary MRI (*n* = 47)				
Sensitivity	37.5% (3/8)	25.0% (2/8)	62.5% (5/8)	62.5% (5/8)
	[8.5–75.5]	[3.2–65.1]	[24.5–91.5]	[24.5–91.5]
Specificity	92.3% (36/39)	87.2% (34/39)	82.1% (32/39)	92.3% (36/39)
	[79.1–98.4]	[72.6–95.7]	[66.5–92.5]	[79.1–98.4]
PPV	50.0% (3/6)	28.6% (2/7)	44.6% (5/12)	62.5% (5/8)
	[11.8–88.2]	[3.7–71.0]	[15.2–72.3]	[24.5–91.5]
NPV	87.8% (36/41)	85.0% (34/40)	91.4% (32/35)	92.3% (36/39)
	[73.8–95.9]	[70.2–94.3]	[76.9–98.2]	[79.1–98.4]
AUC	0.65	0.57	0.73	0.78
	[0.46–0.83]	[0.39–0.75]	[0.53–0.92]	[0.60–0.97]

Data in parentheses are absolute numbers. Data in brackets are 95% confidence intervals

*The additional information of the DW MR images was used to adjust the initial score based on the T2W MR images. If the DW image score was unavailable, the T2W image score remained unchanged

*Abbreviations*: *FOV*, field of view; *T2W*, T2-weighted; *DW*, diffusion-weighted; *PPV*, positive predictive value; *NPV*, negative predictive value; *AUC*, area under the curve

**Fig. 2:**
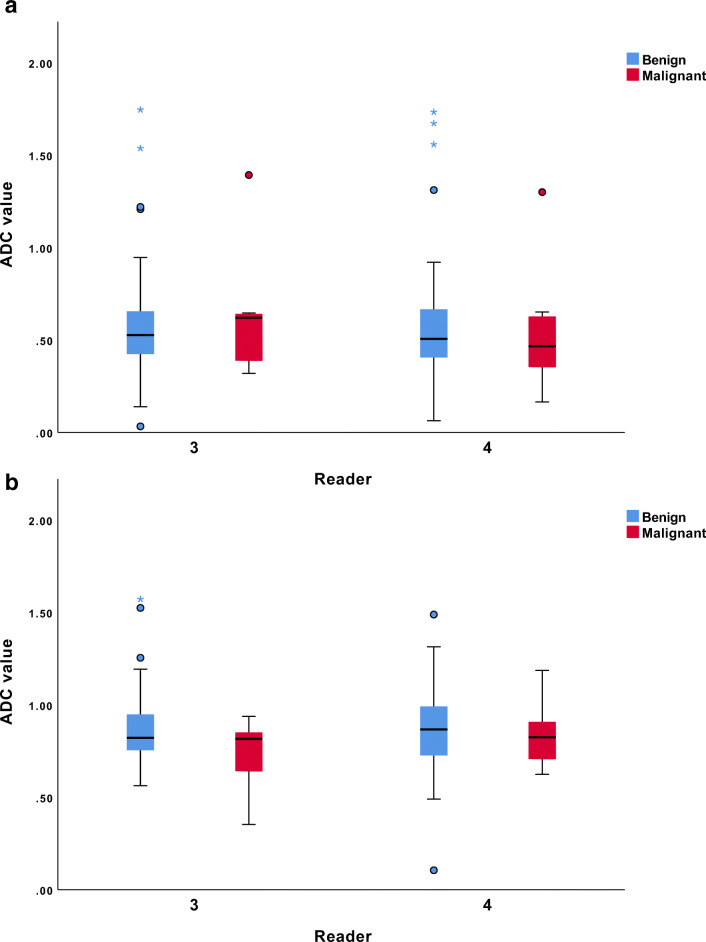
Box plots show the comparison of the mean ADC values (× 10^−3^ mm^2^/s) for benign and malignant lymph nodes. **a** Standard breast MRI: readers 3 and 4. **b** Dedicated axillary MRI: readers 3 and 4

For standard breast MRI, the mean ADC values of benign lymph nodes were 0.608 × 10^−3^ mm^2^/s and 0.611 × 10^−3^ mm^2^/s for readers 3 and 4, respectively. The mean ADC values for malignant lymph nodes were 0.627 × 10^−3^ mm^2^/s and 0.556 × 10^−3^ mm^2^/s for readers 3 and 4, respectively. For both readers, there was no significant difference between the mean ADC value of benign and malignant lymph nodes (reader 3, *p* = 0.40; reader 4, *p* = 0.61) (Fig. 2a). The intraclass correlation coefficient was excellent (0.94). Since two of the standard breast MRI exams had no DW images, the corresponding ADC maps were also unavailable for those MRI exams. Three other ADC maps had poor image quality assessed by the readers. The mean difference in ADC measurement pairs was 0.0092 × 10^−3^ mm^2^/s with 95% limits of agreement of − 0.033 × 10^−3^ and 0.051 × 10^−3^ mm^2^/s.

**Fig. 3:**
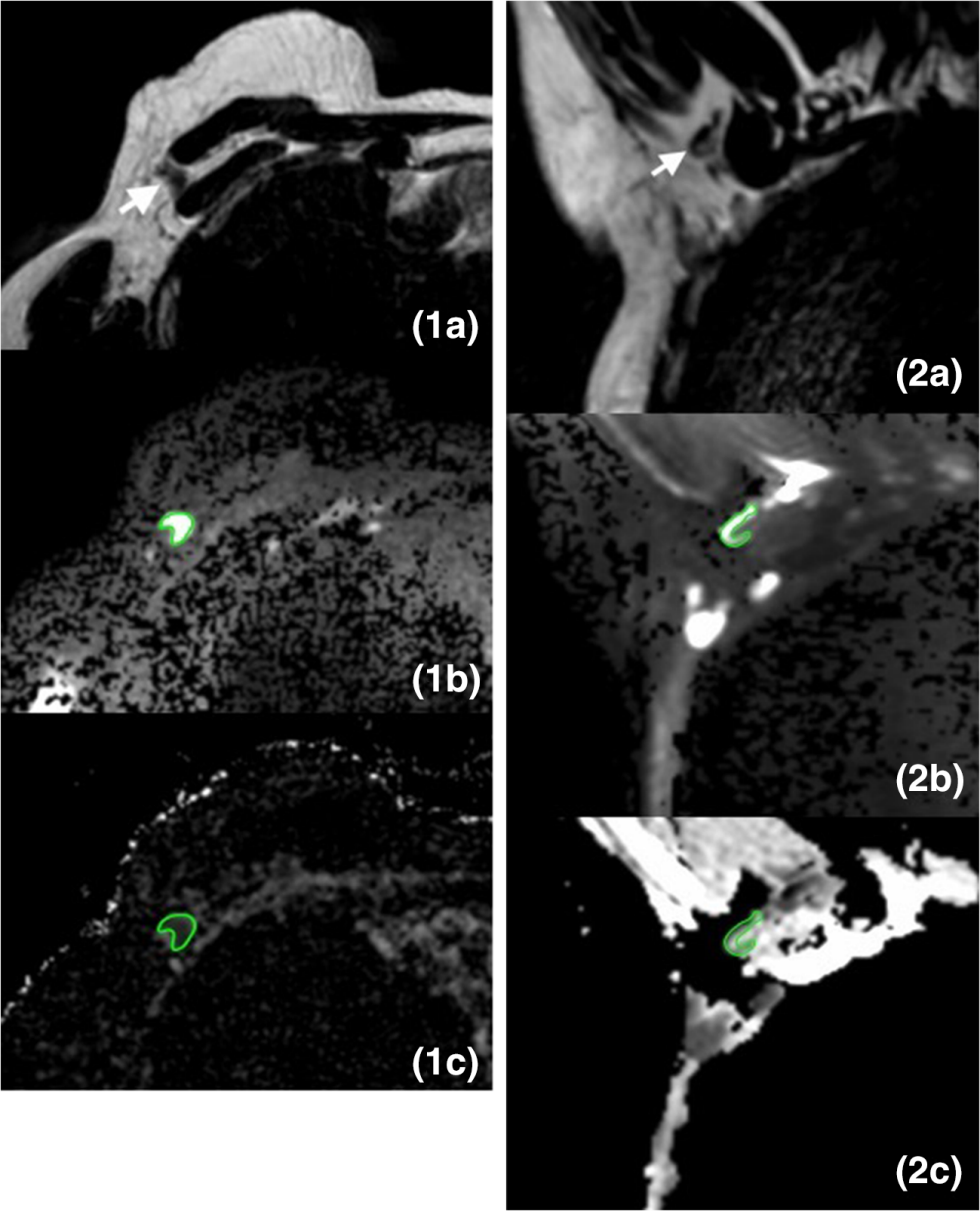
Images of a 27-mm large invasive ductal carcinoma in the right breast of a 64-year-old female patient, which was treated with mastectomy and ALND (pT2N1). The white arrow points to an axillary lymph node. The green delineation shows an axillary lymph node. **1a** Axial T2W breast MR image shows the axillary lymph node with the longest axis. **1b** Axial DW breast MR image (*b*-value = 800 s/mm^2^) shows the same axillary lymph node with relatively high signal intensity. **1c** Axial ADC map of breast MRI shows corresponding lymph node with relatively low signal intensity with an ADC value of 0.646 × 10^−3^ mm^2^/s. **2a** Coronal T2W dedicated axillary MR image shows the axillary lymph node with the longest axis. **2b** Coronal DW dedicated axillary MR image (*b*-value = 800 s/mm^2^) shows the same lymph node with relatively high signal intensity. **2c** Coronal ADC map of dedicated axillary MRI shows corresponding lymph node with relatively low signal intensity with an ADC value of 0.837 × 10^−3^ mm^2^/s

**Fig. 4:**
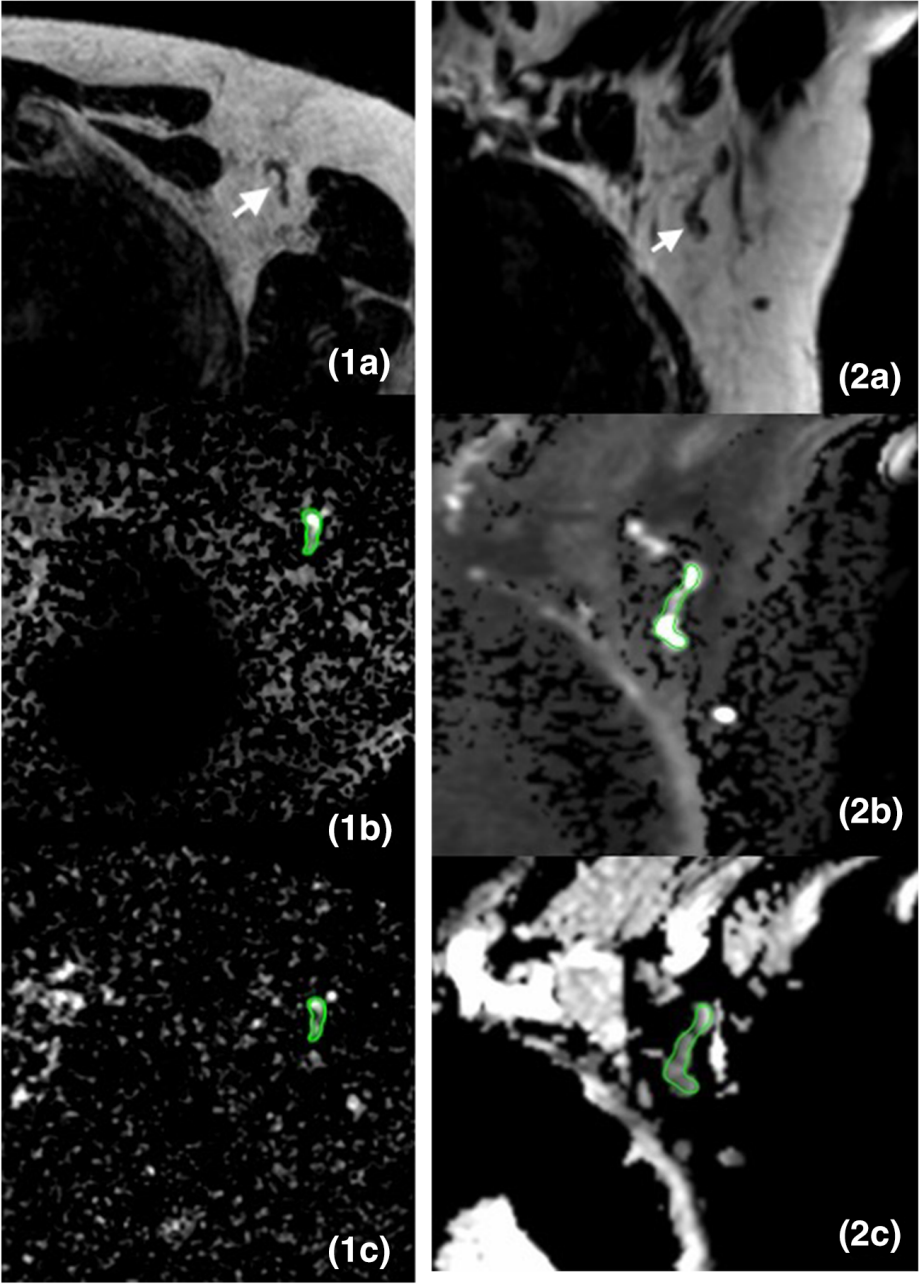
Images of a 24-mm large invasive ductal carcinoma in the left breast of a 76-year-old female patient, which was treated with breast-conserving surgery and SLNB (pT2N0). The white arrow points to an axillary lymph node. The green delineation shows an axillary lymph node. **1a** Axial T2W breast MR image shows the axillary lymph node with the longest axis. **1b** Axial DW breast MR image (*b*-value = 800 s/mm^2^) shows the same axillary lymph node with relatively high signal intensity. **1c** Axial ADC map of breast MRI shows corresponding lymph node with relatively high signal intensity with an ADC value of 0.526 × 10^−3^ mm^2^/s. **2a** Coronal T2W dedicated axillary MR image shows the axillary lymph node with the longest axis. **2b** Coronal DW dedicated axillary MR image (*b*-value = 800 s/mm^2^) shows the same lymph node with relatively high signal intensity. **2c** Coronal ADC map of dedicated axillary MRI shows corresponding lymph node with relatively low signal intensity with an ADC value of 0.940 × 10^−3^ mm^2^/s

For dedicated axillary MRI, the mean ADC values of benign lymph nodes were 0.879 × 10^−3^ mm^2^/s and 0.866 × 10^−3^ mm^2^/s for readers 3 and 4, respectively. The mean ADC values for malignant lymph nodes were 0.727 × 10^−3^ mm^2^/s and 0.838 × 10^−3^ mm^2^/s for readers 3 and 4, respectively. For both readers, there was no significant difference between the mean ADC values of benign and malignant lymph nodes (reader 3, *p* = 0.74; reader 4, *p* = 0.87) (Fig. 2b). The intraclass correlation coefficient was good (0.75). Since one of the dedicated axillary MRI exams had no DW image, the corresponding ADC map was also unavailable. The mean difference in ADC measurement pairs was − 0.0056 × 10^−3^ mm^2^/s with 95% limits of agreement of − 0.053 × 10^−3^ and 0.042 × 10^−3^ mm^2^/s. Examples of an axillary lymph node on T2W image, DW image, and ADC map for standard breast MRI and dedicated axillary MRI are shown in Figs. 3 and 4.

## Discussion

This study showed that the diagnostic performance of unenhanced T2W standard breast MRI with complete FOV of the axillary region is comparable with unenhanced T2W dedicated axillary MRI regarding the assessment of node-negative and node-positive breast cancer. Concerning the diagnostic performance of both MRI exams, especially the relatively high NPV can be used for clinical decision making. For both standard breast MRI and dedicated axillary MRI, DW images and ADC measurements were of no added value. To our knowledge, this is the first study that has compared standard breast MRI and dedicated axillary MRI within a single cohort of breast cancer patients.

In this present study, all breast cancer patients underwent a standard breast MRI for the evaluation of disease extent, to identify multicentric or multifocal disease, or to identify the presence of additional breast lesions. The evaluation of all axillary lymph nodes can be limited if only breast coils are used especially the lymph nodes located in the upper part of the axilla. In this study, only breast MRI exams with complete FOV and sufficient SNR of the axillary region were included to make the comparison with dedicated axillary MRI exams as equal as possible. Dedicated axillary MRI exams may improve the visualization of lymph nodes in axillary levels ΙΙ and ΙΙΙ which may not be identified easily due to the location. However, previous research showed that up to 94% of the SLNs are located in axillary levels Ι and ΙΙ [26–29]. This implies that visualizing axillary level ΙΙΙ, especially in the case of clinically node-negative findings, can be redundant in most cases.

In this study, the unenhanced MR sequence was used since it has been previously described that this sequence has the best anatomical presentation of the lymph nodes based on size and morphology [10, 12, 22]. In addition to the unenhanced T2W sequence, DW imaging has been investigated as an adjunct to help differentiate between benign and malignant lymph nodes. Previously published data about lymph node assessment on unenhanced breast MR imaging showed a sensitivity of 88%, a specificity of 82%, and an accuracy of 85%, and the addition of DW imaging resulted in a sensitivity of 84%, a specificity of 77%, and an accuracy of 80% [22]. The addition of DW imaging was insufficient to improve the diagnostic performance of axillary lymph node assessment, which is in line with our results for both standard breast and dedicated axillary MRI. Further studies evaluating DW imaging showed varying results of sensitivity and specificity of 51.3–94.7% and 90.0–91.8%, respectively [20, 30].

For the quantitative analysis, the ADC values were calculated from the DW imaging. Discordant results have been reported about the ADC values of lymph nodes. Previous research reported similar ADC values for benign and malignant lymph nodes [31], higher values in malignant nodes [32], and lower values in malignant nodes [30, 33, 34]. Our analyses for both standard breast and dedicated axillary MRI showed that the ADC values for benign and malignant lymph nodes are similar. These varying results can be possibly explained by the following: signal intensity on DW imaging can be influenced by lymph node changes like necrotic areas and inflammatory processes. These changes can cause artifacts on the ADC map and therefore not reflect the cellularity of the lymph node. Also, the variety of *b*-value combinations in different studies can significantly affect the ADC value of lymph nodes [35].

In addition to standard breast MRI, a dedicated axillary MRI was performed in a different session by using a surface coil on the patients’ axilla. With the surface coil, the complete axillary region is visualized in a coronal plane. An additional dedicated axillary MR image requires scanning time per axilla and bilateral evaluation to check for asymmetry is not possible. A few studies have evaluated the diagnostic performance of MR imaging with a dedicated axillary surface coil in the preoperative detection of axillary lymph node metastases [11, 12, 15, 36]. These studies reported sensitivity and specificity between 79.0 and 94.6% and 90.0 and 98.5%, respectively [11, 15, 36]. However, these results of dedicated axillary MR imaging are not comparable with those of the present study since contrast-enhanced T1W and T2*W sequence were used for analyses of axillary lymph node metastases.

Recent advances in the medical image analysis field have been made by introducing artificial intelligence for image recognition tasks [37]. Artificial intelligence with methods ranging from radiomics to convolutional neural networks can provide quantitative rather than qualitative imaging data in an automated fashion [37]. With artificial intelligence as a tool to assist radiology image workflow, the imaging assessment can be made more accurate and reproducible [37]. The assessment of axillary lymph nodes by combining the MR images and artificial intelligence can increase the diagnostic outcome. Future research should provide insights into this topic.

Our study has certain limitations. The number of included patients in this study was relatively small, so that potential minor differences between diagnostic performances could have gone undetected. Given the small sample size, further research on this topic is necessary. Also, the statistical analyses were not based on a node-by-node comparison between the visualized lymph nodes and their pathological findings. We assumed that the overall T2W and DWI score of the axilla (benign or malignant) was correlated with the pathology outcome. This was also the case for the identified lymph node with the longest axis on DWI and the corresponding ADC map. Further, two types of MRI systems (1.5 and 3.0 T) were used. However, the varying MR protocols could not be compared due to the small sample size. Finally, nine DW images of breast MRI were inevitably excluded because of movement or susceptibility artifacts, which could have influenced our DW imaging findings for standard breast MRI. A previous study has also suffered from the same issue [22].

In conclusion, the diagnostic performance of T2W standard breast MRI with complete FOV of the axillary region is comparable with that of the T2W dedicated axillary MRI regarding the assessment of node-negative and node-positive breast cancer. Optimization of T2W standard breast MRI protocol by including a complete FOV of the axillary region can, therefore, be recommended in clinical practice.

## Abbreviations

2D: Two-dimensional
3D: Three-dimensional
ADC: Apparent diffusion coefficient
ALND: Axillary lymph node dissection
AUC: Area under the curve
CI: Confidence interval
DW: Diffusion-weighted
FOV: Field of view
IQR: Interquartile range
MRI: Magnetic resonance imaging
NPV: Negative predictive value
NST: Neoadjuvant systemic therapy
PPV: Positive predictive value
ROC: Receiver operating characteristic
ROI: Region of interest
SLNB: Sentinel lymph node biopsy
SNR: Signal-to-noise ratio
T2W: T2-weighted
TE: Echo time
TR: Repetition time
